# Vitamin D/VDR signaling induces miR-27a/b expression in oral lichen planus

**DOI:** 10.1038/s41598-019-57288-9

**Published:** 2020-01-15

**Authors:** Xuejun Ge, Lu Yuan, Jizhen Wei, Tivoli Nguyen, Chenwei Tang, Wang Liao, Ran Li, Fang Yang, Fang Zhang, Bin Zhao, Jie Du

**Affiliations:** 1 Department of Periodontics, Shanxi Medical University School and Hospital of Stomatology, Taiyuan, Shanxi China; 2 Division of Biological Sciences, Department of Medicine, The University of Chicago, Chicago, Illinois USA; 3 Department of Cardiology, Hainan General Hospital, Hainan Clinical Medicine Research Institution, Haikou, China; 4 Department of Oral Medicine, Shanxi Medical University School and Hospital of Stomatology, Taiyuan, Shanxi China; 5 Department of prosthodontics, Shanxi Medical University School and Hospital of Stomatology, Taiyuan, Shanxi China

**Keywords:** Cell biology, Mucositis

## Abstract

MicroRNA-27a/b are small non-coding RNAs which are reported to regulate inflammatory response and cell proliferation. Although some studies have demonstrated that miR-27b is down-regulated in the oral specimens of patients suffering with oral lichen planus (OLP), the molecular mechanism of miR-27b decrease remains a large mystery, and the expression of miR-27a in OLP is not well explored. Here, we demonstrated both miR-27a and miR-27b, compared with healthy controls, were reduced in the oral biopsies, serum and saliva samples derived from OLP patients. The reductions of miR-27a/b were also confirmed in the lipopolysaccharide (LPS)- or activated CD4^+^ T cell-treated human oral keratinocytes (HOKs). Furthermore, we found vitamin D receptor (VDR) binding sites in the promoters of *miR-27a/b* genes and verified this finding. We also tested miR-27a/b levels in the oral epithelium from paricalcitol-treated, vitamin D deficient or *VDR* knockout mice. In the rescue experiments, we confirmed vitamin D and VDR inhibited LPS- or activated CD4^+^ T cell-induced miR-27a/b reductions in HOKs. In sum, our results show that vitamin D/VDR signaling induces miR-27a/b in oral lichen planus.

## Introduction

Oral lichen planus, regarded as a kind of chronic mucocutaneous inflammatory disorder, is considered to be one of the potentially malignant diseases in a common consensus^1^. It affects approximately 2% of the adult population with a higher prevalence in women^2^. Although tremendous investigations have been carried out and much progress has been achieved, the pathogenesis of OLP remains a mystery. It is evident that autoimmune response, infection and mental pressure all contribute to OLP initiation and development^3,4^. In clinic, patients suffering with OLP often show symptoms such as pain, irritation and burning, even in the process of food intake^3^. Histopathologic features of OLP include a typical T cell-infiltrated band in lamina propria; hyperparakeratosis, cytoid bodies and hydropic change in epithelial layer^3^. So far, OLP seems to be an incurable disease and most of clinical work focuses on inflammation control and symptoms reduction^3^. Therefore, it is urgent to investigate the pathogenesis of OLP and look for a curative way for it.

MicroRNAs (miRNAs) are a group of small, noncoding RNAs, which suppress target mRNAs expression through interacting with 3′UTR of them^5^. In published studies, miRNAs are reported to exert their biological functions in inflammation, metabolism, and development^6–11^. In the field of OLP, several studies have regarded miRNAs as biomarkers for disease progression and malignant transformation^12,13^. Cellular miRNA-27 is expressed throughout a variety of tissues and cell types ubiquitously and is highly conserved^14,15^. To date, investigations of miR-27′s functions are mostly focused on the immune system and cell proliferation^10,11,14–17^. In the context of OLP, miR-27b is reported to be down-regulated in oral biopsies of OLP patients by a couple of studies^2,18^. However, the expression of miR-27a in OLP is not clear, and the mechanism of miR-27a/b reductions is not well explained. Given this, exploitation of miR-27a/b in OLP will help us to better understand the cause of OLP.

Vitamin D, a pleiotropic hormone, plays critical roles in a broad range of biologic activities^19^. The active form of vitamin D, synthesized in the kidney, is referred as 1,25-dihydroxyvitamin D [1,25(OH)_2_D_3_]^20^. 1,25(OH)_2_D_3_ takes its regulatory actions via interacting with vitamin D receptor (VDR)^21^. Vitamin D/VDR signaling is confirmed to possess regulatory functions in inflammatory diseases^22^. We have reported vitamin D/VDR signaling is suppressed in the oral biopsies or serum samples derived from OLP patients^23,24^. In addition, previous studies have also demonstrated that this signaling suppresses miR-802 and hypoxia-inducible factor-1α (HIF-1α) to ameliorate apoptosis and inflammatory reaction in HOKs^25,26^, indicating its protective roles in OLP. In this study, we confirmed that miR-27a/b are down-regulated in oral biopsies, serum and saliva samples of OLP patients, and vitamin D/VDR signaling can induce miR-27a/b expression in OLP.

## Methods and Materials

### Human samples

Buccal mucosal biopsies, blood and saliva samples were collected from healthy individuals and OLP patients at the Hospital of Stomatology affiliated with Shanxi Medical University. Participants who underwent retained wisdom teeth extraction without any visible buccal inflammation were classified as healthy controls. OLP identification and inclusion criteria were set up based on the modified World Health Organization (WHO) diagnostic criteria^27,28^. All participants involved in this study signed the written informed consent. This investigation was approved by the Ethical Committee of Shanxi Medical University. All methods were performed in accordance with the relevant guidelines and regulations. More information on OLP patients was provided in previous studies^23^.

### Animal studies

8-week-old C57BL/6 mice with wildtype background were chosen experimentally for this study. Wildtype mice were administrated with vitamin D analog paricalcitol (300 ng/kg) daily for one week by intraperitoneal injection. For vitamin D deficiency model establishment, mice were placed in a dark room immediately following weaning and fed with a vitamin D deficient or normal diet for eight weeks as described before^29^. VDR−/− mice were generated in term of the previous method^30^. Oral epithelial cells from mice were collected to isolate proteins and miRNAs. All protocols of animal studies were approved by the Ethical Committee of Shanxi Medical University. All methods were performed in accordance with the relevant guidelines and regulations.

### Cell culture

Human oral keratinocytes (HOKs) were placed in 6-well plate and cultured with oral keratinocyte medium containing 10% fetal bovine serum (FBS) and 1% penicillin/streptomycin (P/S). Two types of cell models were employed to mimic OLP *in vitro*. For the first, HOKs were challenged by LPS (100 ng/ml). For the second, the supernatants from T cells culture, which were stimulated with anti-CD3/28, were added into the HOKs culture medium at a 30% final volumetric concentration^25^. For time course-dependent experiments, HOKs were stimulated by LPS or activated CD4^+^ T cells for 0, 4, 8, 12, 24 hours, respectively. For the rest of cell models, unless otherwise specified, HOKs were challenged for 24 hours. In another experiment, HOKs were transfected with VDR or control plasmids (4 µg) for 36 hours or pretreated with 1,25(OH)_2_D_3_ (20 nM) for 12 hours before LPS or activated CD4^+^ T cells treatments. VDR or empty plasmids were transfected by Lipofectamine 2000 (Thermo Fisher Scientific, Waltham, MA). T cell isolation and stimulation were carried out as described before^25^.

### Oral mucosal epithelium isolation

Oral buccal tissues from human and mice were digested with 0.25% dispase II in cold room for 12 hours. Separation of epithelium and lamina propria was completed using muscle forceps as reported^31^.

### Western blot

Western blot analyses were used as mentioned previously^32^. In brief, cells or tissues were dissolved with laemmli buffer, followed by 5-min incubation at 95 °C. Whole cell lysates were separated by SDS-PAGE gel and then transferred onto PVDF membranes. The first antibodies were used to incubate membranes overnight in cold room, followed by one-hour secondary antibody treatment at room temperature. The bands were visualized using an ECL kit (Thermo Fisher Scientific). VDR (sc-13133) and β-actin (sc-47778) antibodies were both from Santa Cruz Biotechnology (Dallas, TX, USA).

### Real-time PCR

Total RNAs from HOKs or oral buccal epithelium were extracted by TRIzol Reagent (Invitrogen, Carlsbad, CA) and mRNAs were purified. The first strand cDNAs were synthesized with PrimeScript RT Reagent Kit (TaKaRa, Mountain View, CA) and real-time PCRs were performed with a SYBR Premix Ex Kit (TaKaRa). miRNAs from tissues and cells were isolated with miRNA isolation kit (Life Technologies), and the circulating miRNAs from serum and saliva were obtained with mirVana PARIS kit (Life Technologies) according to the manufacturer’s instructions. cDNA synthesis and real-time PCR were completed using either a specific miRNA First-strand cDNA Synthesis Kit (Aidlab Biotechnologies, Beijing, China) or an miRNA Real-time PCR Assay Kit (Aidlab Biotechnologies) accordingly. Relative amounts of transcripts were analyzed by the 2^−ΔΔCt^ formula. For circulating miRNA samples, the same amount of exogenous cel-miR-39 was added before miRNA extraction and served as normalization. Sequences of PCR primers were shown in Table 1.

**Table 1:** Primer sequences involved in this work.

Primer name	Forward(5′-3′)	Reverse(5′-3′)
Hsa-mir-27a	TTCACAGTGGCTAAGTTCCGC	
Hsa-mir-27b	TTCACAGTGGCTAAGTTCTGC	
Has-miR-16	TAGCAGCACGTAAATATTGGCG	
Mmu-mir-27a	TTCACAGTGGCTAAGTTCCGC	
Mmu-mir-27b	TTCACAGTGGCTAAGTTCTGC	
U6	GATGACACGCAAATTCGTGAA	
miR-27a ChIP site1	GATGGAGAGGAGAGATCGTGC	GAGCCAGTGTACACAAACCAAC
miR-27a ChIP site2	GCCTGGCCTTTTATTGTTTG	GGTGGTGGGTGCCTGTAA
miR-27a ChIP site3	CCCAGTTCACACGATTCTCC	CATGGCGAAACTCGGTCT
miR-27b ChIP	TGCCACAAGAAGGCTATTATCCA	CTGCTCTCATATCAGCACTTCC
hsa-let-7a-2 ChIP	CCTGCCTTGTGTCCCATTCATAAG	GTCTTCTGCTACTAGATGCTCACT
hVDR	GACTTTGACCGGAACGTGCCC	CATCATGCCGATGTCCACACA
hTNFα	CCTCTCTCTAATCAGCCCTCTG	GAGGACCTGGGAGTAGATGAG
hIL-6	TGAGGAGACTTGCCTGGTGA	GTTGGGTCAGGGGTGGTTAT
hIFNγ	TGAACATGATGGATCGTTGG	CATTCACTTTGCTGGCAGTG
hSNAP25	ACCAGTTGGCTGATGAGTCG	CAAAGTCCTGATACCAGCATCTT
hTXN2	CTGGTGGCCTGACTGTAACAC	TGACCACTCGGTCTTGAAAGT
hGADPH	ACCACAGTCCATGCCATCAC	TCCACCACCCTGTTGCTGTA

### Chromatin immunoprecipitation (ChIP) assays

ChIP assays were carried out using a commercial kit according to the manufacture instruction. In brief, HOK cells transfected with VDR or empty plasmids were fixed in 1% formaldehyde and then treated with glycine for neutralization. Cell lysates were sonicated and precipitated with the help of antibody (VDR or IgG) and protein A agarose. After elution and a series of washes, samples were quantified by qPCR. The primers for ChIP assay were provided in Table 1.

### Statistical analysis

Data are shown as means ± SD. 2 groups data were analyzed using 2-tailed Student’s *t* test, and multiple groups data were analyzed by one-way ANOVA. *P* < 0.05 was considered statistically significant.

## Results

### miR-27a/b are down-regulated in OLP patients and OLP models

**Figure 1:**
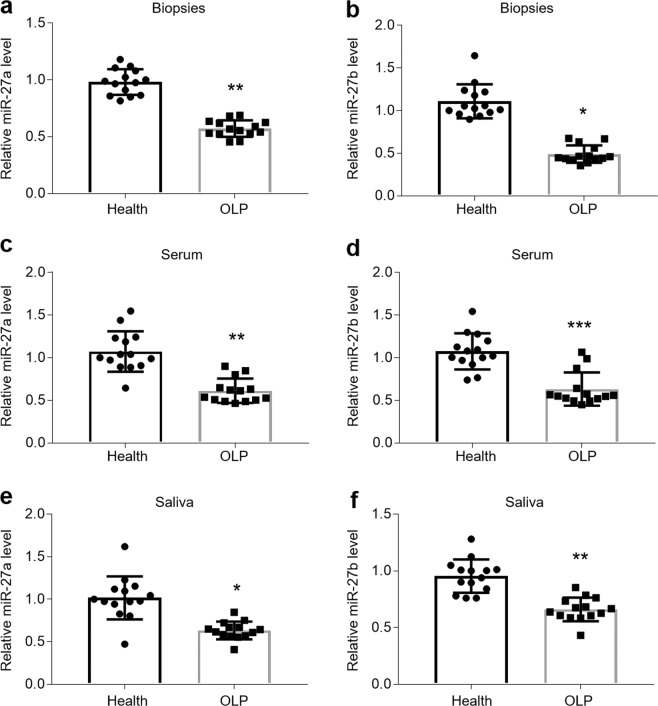
miR-27a/b levels are down-regulated in OLP patients. (**a**,**b**) The levels of miR-27a (**a**) and miR-27b (**b**) in human oral epithelia measured by qPCR. (**c**,**d**) The expression of miR-27a (**c**) and miR-27b (**d**) in human serum monitored by qPCR. (**e**,**f**) miR-27a (**e**) and miR-27b (**f**) status in human saliva detected by real-time PCR. *P < 0.05, **P < 0.01, ***P < 0.001 vs. corresponding healthy controls; n = 14.

**Figure 2:**
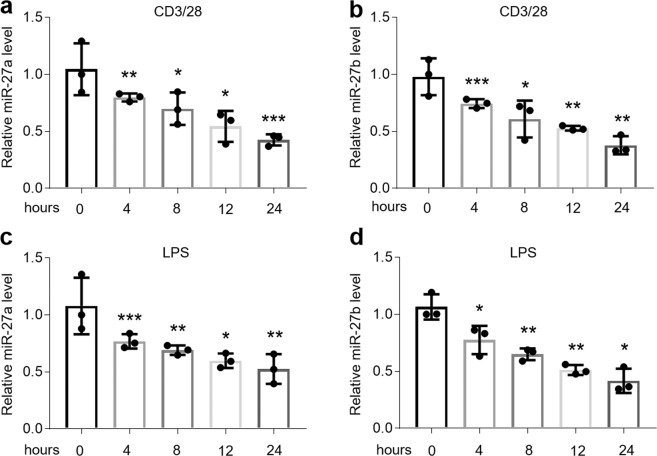
miR-27a/b levels are reduced in LPS- or activated CD4^+^ T cell-treated HOKs. (a and b) miR-27a (a) and miR-27b (**b**) levels in activated CD4^+^ T cell-treated HOKs determined by qPCR at different time points as indicated. (**c**,**d**) Real-time PCR quantification of miR-27a (**c**) and miR-27b (**d**) levels in HOKs treated with time course-dependent LPS. *P < 0.05, **P < 0.01, ***P < 0.001 vs. corresponding control; n = 3.

Previous studies have demonstrated that miR-27b shows a significant decrease in oral biopsies of OLP patients^2,12,18^. To confirm the miR-27b expression and exploit miR-27a in OLP, we collected oral epithelium, blood and saliva samples from individuals to investigate miR-27a/b levels. As shown in Fig. 1, miR-27a/b were decreased in the epithelial layer of oral biopsies (Fig. 1a,b) of OLP patients. Furthermore, circulating miR-27a/b levels of serum were lower in OLP group than those in health controls (Fig. 1c,d), and so were they in saliva samples (Fig. 1e,f). As autoimmune response and infection are both able to induce OLP, to better mimic it *in vitro*, we treated HOKs with LPS or activated CD4^+^ T cell supernatants to establish OLP models. As expected, miR-27a/b were down-regulated in the two cell models in a time-dependent manner (Fig. 2a–d). TNFα, IL-6 and IFNγ, selected as positive controls, were also enhanced in the presence of LPS or activated CD4^+^ T cell supernatants (Supplemental Fig. 1a–f). Importantly, LPS or activated CD4^+^ T cell supernatants had no effects on miR-16 which is used for a negative control (Supplemental Fig. 2a,b).

### VDR binds to its elements in the promoters of *miR-27a/b* to enhance their expression in HOKs

**Figure 3:**
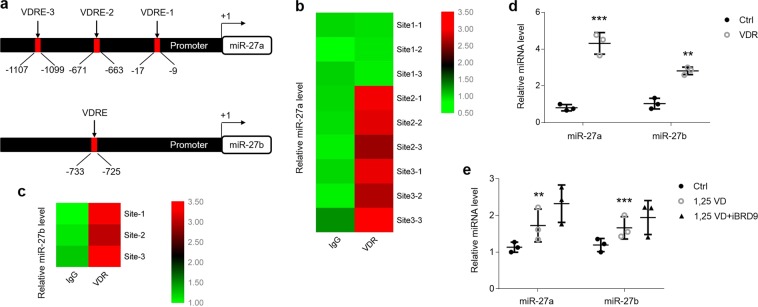
Vitamin D and VDR promote miR-27a/b expression in HOKs. (**a**) Schematic illustration of VDR binding sites in *miR-27a/b* promoters. (**b**) ChIP analysis indicating the up-regulation of VDR binding sites in *miR-27a* in HOKs transfected with VDR plasmids after IgG or VDR antibodies precipitation as indicated. Sites 1–3 mean VDREs 1–3, correspondingly. Bar demonstrates log_2_ fold change, n = 3 for each site. (**c**) ChIP analysis indicating the up-regulation of VDR binding site in *miR-27b* in HOKs transfected with VDR plasmids after IgG or VDR antibodies treatment. Bar demonstrates log_2_ fold change, n = 3 for this site. (**d**) Real-time PCR test of miR-27a/b levels in HOKs transfected with or without VDR plasmids. (**e**) Real-time PCR determination of miR-27a/b in HOKs with different treatments as indicated. **P < 0.01, ***P < 0.001 vs. corresponding control; n = 3. Ctrl, control; 1,25VD, 1,25(OH)_2_D_3_.

To determine the mechanism of miR-27a/b decreases in OLP, we examined the promoters of *miR-27a/b*, where we found VDR elements (VDREs) (Fig. 3a). It seems there are three putative VDR binding sites (1–3) in the promoter of *miR-27a* (Supplemental Fig. 3a), but our ChIP data showed that only VDRE-2 and VDRE-3 comprise the authentic binding sites for VDR (Fig. 3b and Supplemental Fig. 3c). Furthermore, compared with the mild increase in HOKs transfected with empty plasmids, VDR overexpression largely enhanced the combination of VDR and VDRE (Supplemental Fig. 3d,e). What is more, there is a VDRE in the promoter of *miR-27b* (Supplemental Fig. 3b), which was confirmed by ChIP assay in HOKs transfected with or without VDR plasmids (Fig. 3c and Supplemental Fig. 3f). To further verify the role of VDR in miR-27a/b induction, we transfected VDR plasmids into HOKs and tested miR-27a/b inductions. As shown in Fig. 3, miR-27a/b levels were highly increased in the presence of VDR plasmids (Fig. 3d). Hsa-let-7a-2, a positive control for VDRE investigation^33^, also displayed higher expression in HOKs after VDR overexpression (Supplemental Fig. 3g). SNAP25 and TXN2 are two target genes of miR-27a/b^14^, and we next sought to explore the expression of them. Accompanied with miR-27a/b increases, VDR overexpression down-regulated SNAP25 and TXN2 levels (Supplemental Fig. 3h). Vitamin D is reported to activate VDR in most kinds of cells to exert its biological functions^21^. To this end, we added 1,25(OH)_2_D_3_ into HOKs culture medium in this investigation. As displayed, vitamin D mildly up-regulated miR-27a/b status (Fig. 3e). Pharmacological inhibition of bromodomain-containing protein 9 (iBRD9) is reported to enhance VDR’s biological function^34^, and our data showed that iBRD9 facilitated vitamin D to increase miR-27a/b expression (Fig. 3e).

### Vitamin D/VDR signaling regulates miR-27a/b expression in oral epithelial cells of mice

**Figure 4:**
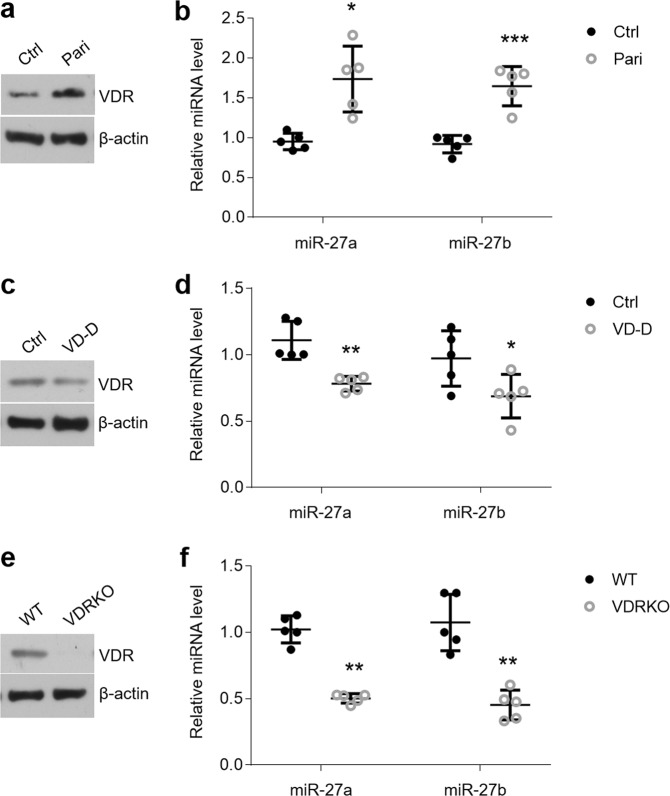
miR-27a/b expression is mediated by vitamin D and VDR *in vivo*. (**a**,**b**) VDR expression (**a**) and miR-27a/b levels (**b**) of oral epithelial cells were measured by western blot and qPCR respectively in paricalcitol-treated mice. (**c**,**d**) Western blot and qPCR showing VDR (**c**) and miR-27a/b (**d**) status of oral epithelium in vitamin D deficient mice. (**e**,**f**) Western blot and qPCR displaying VDR expression (**e**) and miR-27a/b levels (**f**) of oral epithelial layer in *VDR* knockout mice. *P < 0.05, **P < 0.01, ***P < 0.001 vs. corresponding control or WT; n = 5. Ctrl, control; pari, paricalcitol; VDRKO, VDR knockout; VD-D, vitamin D-deficiency; WT, wildtype.

To further detect the effect of vitamin D/VDR signaling on miR-27a/b *in vivo*, we treated C57BL/6 mice with paricalcitol. Accompanied with increase of VDR expression, miR-27a/b levels were up-regulated in the oral epithelial cells (Fig. 4a,b). In contrast, expression of miR-27a/b of oral epithelium was down-regulated in vitamin D deficient or *VDR* knockout mice, which showed either VDR decrease or VDR deletion (Fig. 4c–f). These results provide evidence for the mediation of vitamin D/VDR signaling on miR-27a/b *in vivo*.

### Inhibition of vitamin D/VDR signaling results in miR-27a/b decreases in OLP

**Figure 5:**
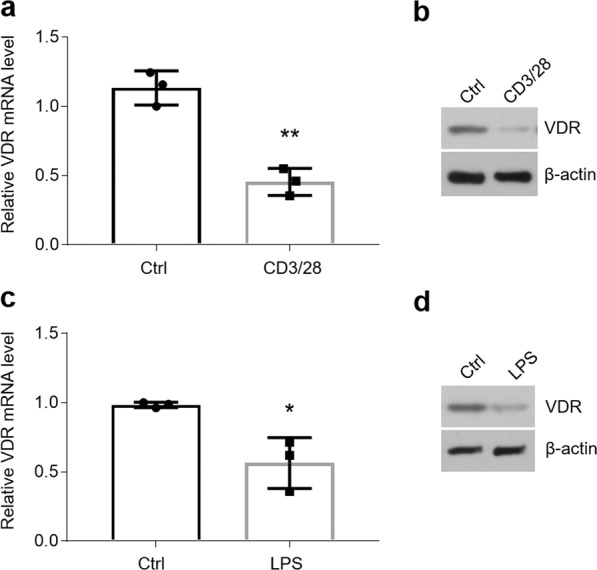
VDR levels show significant decreases in OLP cell models. (a and b) VDR mRNA (**a**) or protein (**b**) levels in HOKs treated with or without activated CD4^+^ T cells tested by qPCR or western blot. (**c**,**d**) VDR mRNA (**c**) or protein (**d**) levels in HOKs in the presence or absence of LPS treatment detected by qPCR or western blot. *P < 0.05, **P < 0.01 vs. corresponding control; n = 3. Ctrl, control.

**Figure 6:**
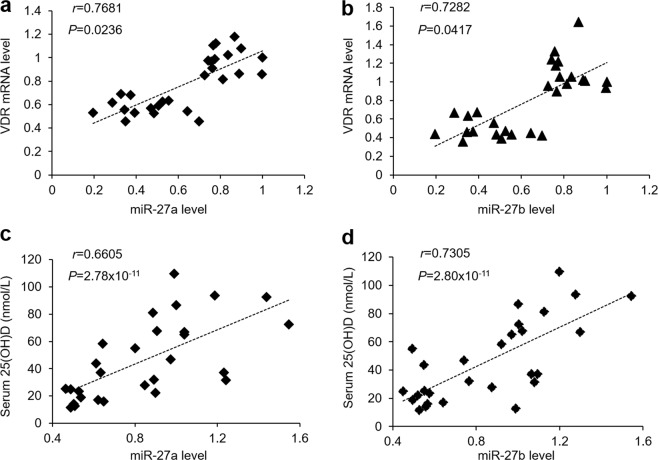
Vitamin D/VDR and miR-27a/b show good positive correlations in OLP patients and healthy controls. (**a**,**b**) Correlations of VDR and miR-27a (**a**)/miR-27b (**b**) in human oral epithelial cells from OLP patients and healthy controls (*r* = 0.7681, *P* = 0.0236, Spearman’s correlation test for miR-27a; *r* = 0.7282, *P* = 0.0417, Spearman’s correlation test for miR-27b), n = 14. (**c**,**d**) Correlations of 25(OH)D and miR-27a (**c**)/miR-27b (**d**) in human serum derived from OLP patients and healthy controls (*r* = 0.6605, *P* = 2.78 × 10^-11^, Spearman’s correlation test for miR-27a; *r* = 0.7305, *P* = 2.80 × 10^−11^, Spearman’s correlation test for miR-27b), n = 14.

Our previous studies have indicated that status of VDR in biopsies and vitamin D in serum are down-regulated in OLP patients^23,24^, which indicates the cause of miR-27a/b decreases in OLP might be, at least in part, due to vitamin D/VDR signaling suppression. In accordant with the results regarding human samples, we tested VDR expression in the two kinds of cell models and found their levels were compromised in HOKs with LPS or activated CD4^+^ T cell treatment (Fig. 5a–d). Accordingly, positive correlations were observed between VDR and miR-27a/b in oral epithelial cells obtained from OLP patients and controls (Fig. 6a,b [*r* = 0.7681, *P* = 0.0236, Spearman’s correlation test for miR-27a] and [*r* = 0.7282, *P* = 0.0417, Spearman’s correlation test for miR-27b]), and 25(OH)D and miR-27a/b in serum from participants also showed good correlations (Fig. 6c,d [*r* = 0.6605, *P* = 2.78 × 10^−11^, Spearman’s correlation test for miR-27a] and [*r* = 0.7305, *P* = 2.80 × 10^−11^, Spearman’s correlation test for miR-27b]).

**Figure 7:**
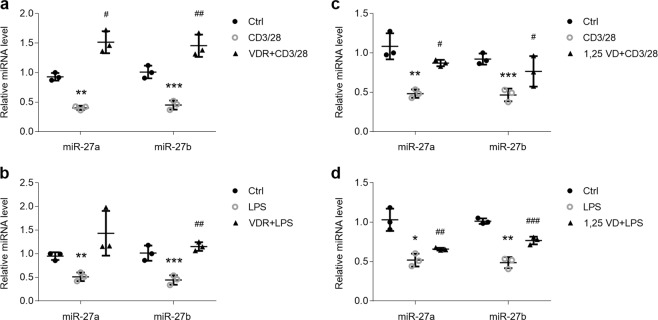
Vitamin D and VDR increase miR-27a/b in OLP cell models. (**a**,**b**) Real-time PCR showing miR-27a/b levels in HOKs treated with activated CD4^+^ T cells (**a**) or LPS (**b**) with or without VDR plasmids transfection as indicated. (**c**,**d**) Real-time PCR indicating miR-27a/b expression in HOKs treated with activated CD4^+^ T cells (**c**) or LPS (**d**) in the presence or absence of 1,25 VD. *P < 0.05, **P < 0.01, ***P < 0.001 vs. corresponding control; ^#^P < 0.05, ^##^P < 0.01, ^###^P < 0.001 vs CD3/28 or LPS group; n = 3. Ctrl, control; 1,25VD, 1,25(OH)_2_D_3_.

If suppression of vitamin D/VDR signaling leads to miR-27a/b decreases in OLP, we hypothesized that vitamin D or VDR treatment would be likely to rescue them. To investigate this hypothesis, we pretreated HOKs with vitamin D or VDR plasmids before LPS or activated CD4^+^ T cell challenge. As shown, VDR plasmids transfection reversed miR-27a/b decreases in the presence of activated CD4^+^ T cell or LPS in HOKs (Fig. 7a,b), and so did 1,25(OH)_2_D_3_ pretreatment (Fig. 7c,d). Moreover, VDR overexpression also ameliorated miR-27a/b decreases in cell culture medium (Supplemental Fig. 4a,b). Accordantly, vitamin D/VDR signaling attenuated TNFα productions in HOKs with LPS or activated CD4^+^ T cell treatment (Supplemental Fig. 4c–f). Together, these data manifest that vitamin D/VDR signaling blockage is one of the reasons of miR-27a/b reductions in OLP.

## Discussion

In this study, we collected oral biopsies, serum and saliva samples from both OLP patients and healthy participants to detect miR-27a/b expression. Compared with healthy controls, miR-27a/b levels were consistently compromised in the three types of samples from OLP patients. These findings are in agreement with previous notes that miR-27b is down-regulated in oral biopsies obtained from OLP patients^2,18^. Importantly, we further investigated miR-27a/b status in OLP cell models by employing LPS treatment to mimic infection conditions in HOKs or adopting activated CD4^+^ T cell supernatants to simulate the microenvironment of autoimmune response. Consistent with the observations found in human samples, miR-27a/b levels showed robust decreases in the two kinds of cell models, providing compelling evidence for our findings.

The major biological activities of miR-27a and miR-27b in bodies are demonstrated to be associated with immune response, cell proliferation and development. In HeLa cells, miR-27 targets SNAP25 and TXN2 to block adenovirus infection^14^. Earlier reports have claimed that miR-27 suppresses inflammatory responses by regulating T-cell functions and cytokines productions^10,15^. On the contrary, miR-27 overexpression has the ability of impairing Treg differentiation^17^. For cell proliferation, miR-27 is demonstrated to promote osteosarcoma cell growth and accelerate chondrogenic differentiation^16,35^, while other studies identify miR-27 as a tumor suppressor in renal cell carcinoma^36^. These inconsistent observations create an unclear understanding regarding the function of miR-27. In the field of OLP, some investigations have suggested that miR-27b targets PLK2 to promote oral keratinocytes proliferation^37^. Due to the inflammatory conditions, miR-27a/b’s functions may be largely involved in immune response in the context of OLP. This hypothesis requires further investigations in our following project. Herein, we focused on the mechanism of miR-27a/b reductions in OLP.

Our recent data have indicated that vitamin D/VDR signaling plays a protective role in OLP by inhibiting cytokines secretion and apoptosis in oral keratinocytes^25,26^. In the current study, we located VDR binding sites in the promoter regions of *miR-27a/b*, implying vitamin D/VDR signaling possesses the potential of inducing miR-27a/b expression. We then carried out ChIP assays to confirm that VDR enhances miR-27a/b transcripts via binding with VDREs. Additionally, VDR plasmids transfection, but not 1,25(OH)_2_D_3_ treatment, considerably improved miR-27a/b yields in HOKs, indicating the primary role of VDR in the vitamin D/VDR signaling. This observation is accordant with previous reports which note that VDR is a key modulator of β cell survival, stromal reprogramming and liver fibrosis^34,38,39^. Since some studies have reported the biological functions of vitamin D are limited in several fields^40^, we suggest more attentions should be placed on VDR itself rather than the vitamin D hormone. Does vitamin D/VDR signaling regulate miR-27a/b transcripts in mice? To answer this question, we established three kinds of models. Our data suggested that vitamin D treatment raised miR-27a/b expression in oral epithelial cells of mice, whereas vitamin D deficiency or *VDR* deletion decreased them. These cell line and mouse data together identify a key role of oral epithelial vitamin D/VDR signaling in the mediation of miR-27a/b expression.

We have demonstrated that VDR levels of oral epithelium are down-regulated by approximately 50% and the 25(OH)D status of serum shows a > 50% decrease in OLP patients in early explorations^23,24^. Consistent with the human data, we further showed a ~50% VDR decrease in OLP cell models. Based on these results, we drew a conclusion that vitamin D/VDR signaling suppression contributes to miR-27a/b decreases in OLP. Importantly, good positive correlations between VDR/25(OH)D and miR-27a/b were found in oral specimens or serum from OLP patients. Due to the lack of well-established OLP animal models, we can not investigate the correlation of VDR and miR-27a/b under diseased conditions in mice.

Vitamin D or VDR overexpression has been proven to perform its regulatory functions in oral epithelium of OLP by impeding nuclear factor-κB (NF-κB) pathway or by inducing von Hippel-Lindau (VHL)^25,26^. In this work, we found that either VDR plasmids transfection or vitamin D treatment attenuated LPS or activated CD4^+^ T cell supernatant-induced miR-27a/b decreases in OLP cell models, proposing a notion that vitamin D/VDR signaling may control miR-27a/b expression to exert its physiological actions in OLP and expand the regulatory networks of vitamin D/VDR signaling.

In conclusion, this work indicates vitamin D/VDR signaling accelerates miR-27a/b expression in OLP. Upon binding with VDREs, vitamin D facilitates VDR to improve miR-27a/b transcripts in oral epithelial cells. Thus, miR-27a/b reductions in OLP may be due, at least in part, to vitamin D/VDR signaling suppression. Although the mechanism of miR-27a/b reductions has been elaborated, the roles of miR-27a/b in OLP development still require further investigations to better understand the pathogenies of this disease.

## Supplementary information

Supplementary information
