# Supplementary material for: Vitamin D/VDR signaling induces miR-27a/b expression in oral lichen planus
Source: Sci Rep. 2020 Jan 15;10:301. doi: 10.1038/s41598-019-57288-9 (PMC6962379; doi:10.1038/s41598-019-57288-9)
Supplement: Supplementary file 1 — Supplementary information [file 41598_2019_57288_MOESM1_ESM.pdf]

Vitamin D/VDR signaling induces miR-27a/b expression in oral lichen planus

Xuejun Ge[1], Lu Yuan[1], Jizhen Wei[1], Tivoli Nguyen[2], Chenwei Tang[2], Wang Liao[3], Ran Li[4], Fang Yang[1], Fang Zhang[4], Bin Zhao[4,5], Jie Du[4*]

[1]Department of Periodontics, Shanxi Medical University School and Hospital of Stomatology, Taiyuan, Shanxi, China.

[2]Division of Biological Sciences, Department of Medicine, The University of Chicago, Chicago, Illinois, USA.

[3]Department of Cardiology, Hainan General Hospital, Hainan Clinical Medicine Research Institution, Haikou, China.

[4]Department of Oral Medicine, Shanxi Medical University School and Hospital of Stomatology, Taiyuan, Shanxi, China.

[5]Department of prosthodontics, Shanxi Medical University School and Hospital of Stomatology, Taiyuan, Shanxi, China.

*Corresponding author: Jie Du, D.D.S., Ph.D., Department of Oral Medicine, Shanxi Medical University School and Hospital of Stomatology, NO. 56 Xinjian South Road, Taiyuan, Shanxi, 030001, China (email: dj1243@hotmail.com)

Supplemental figures

Supplemental figure 1

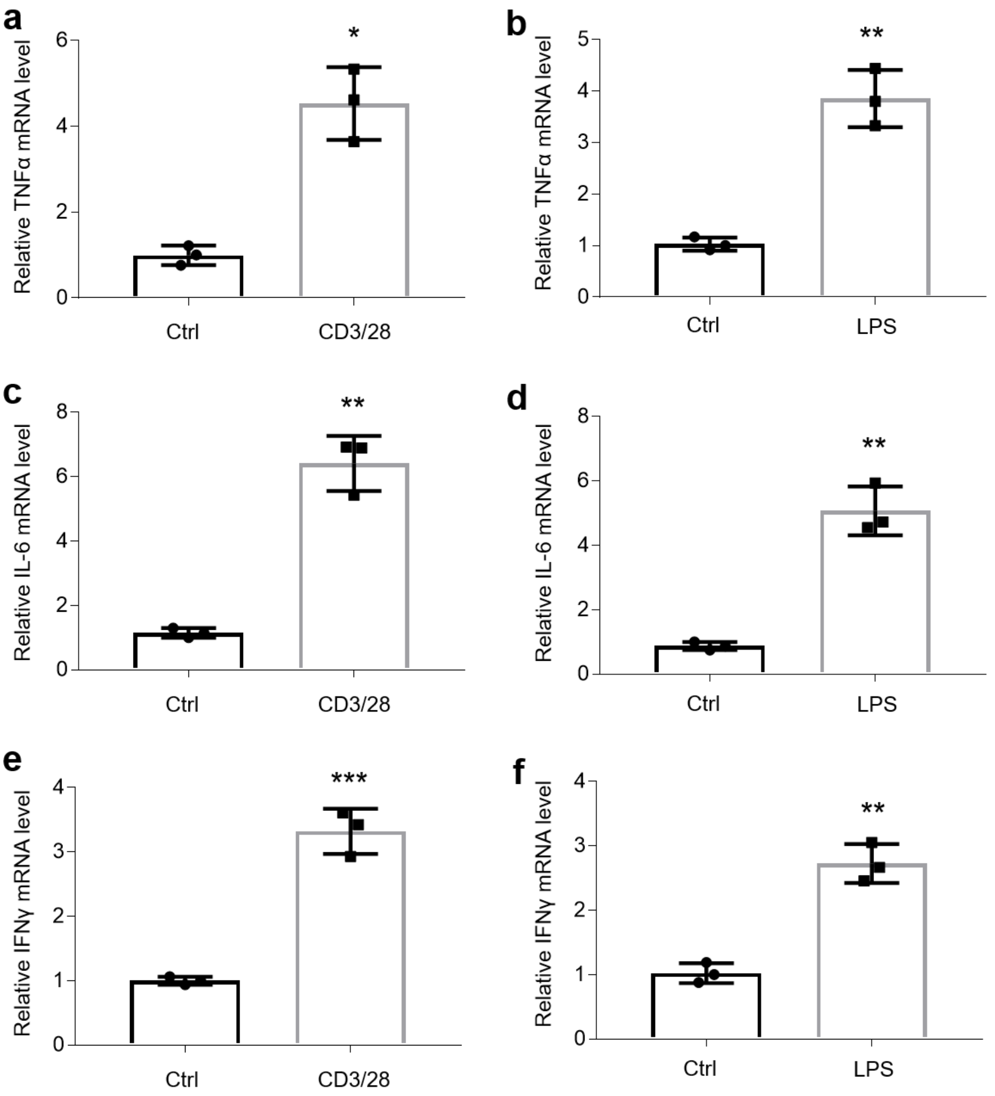

Supplemental Figure 1. Cytokines levels are up-regulated in OLP cell models. (a and b) The levels of TNFα in activated $CD4^+$ T cell (a)- or LPS (b)-treated HOKs determined by qPCR. (c and d) IL-6 expression in activated $CD4^+$ T cell (c)- or LPS (d)-changed HOKs measured by qPCR. (e and f) IFNγ status in activated $CD4^+$ T cell (e)- or LPS (f)-stimulated HOKs detected by real-time PCR. *$P < 0.05$, **$P < 0.01$, ***$P < 0.001$ vs. corresponding control; n = 3. Ctrl, control.

Supplemental figure 2

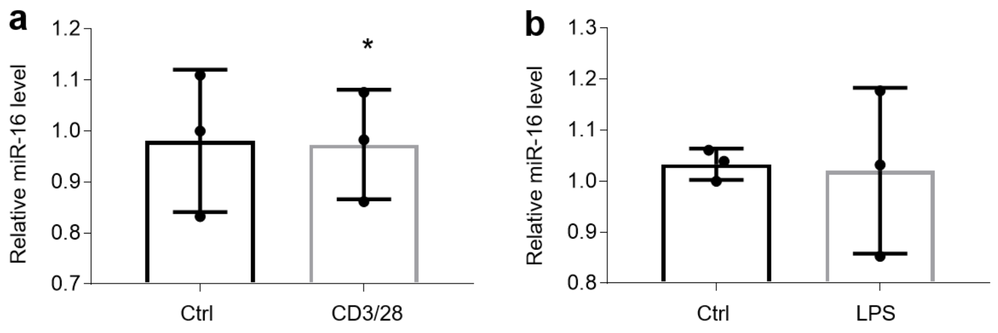

Supplemental Figure 2. LPS and activated CD4$^+$ T cell have no effects on miR-16 levels in HOKs. (a and b) miR-16 expression in activated CD4$^+$ T cell (a)- or LPS (b)-treated HOKs tested by qPCR. *P < 0.05 vs. corresponding control; n = 3. Ctrl, control.

Supplemental figure 3

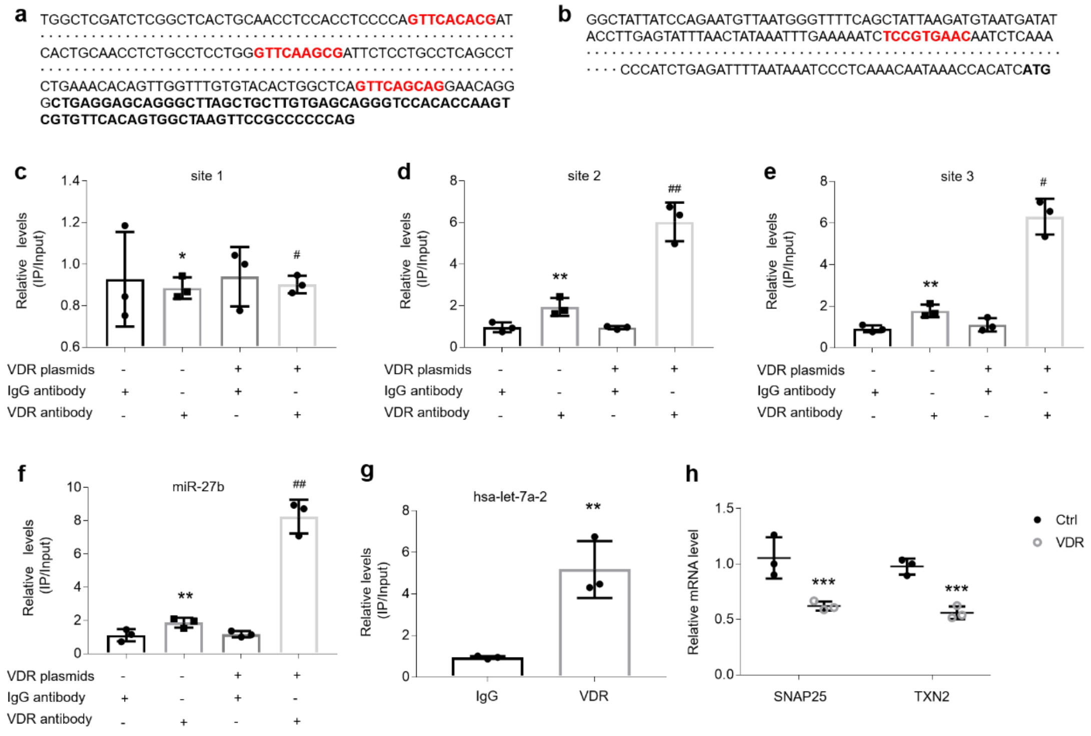

Supplemental figure 3. VDR promotes its downstream genes expression in HOKs. (a) Three putative VDR binding sites (red) in the promoter of human *miR-27a* gene. Nucleotides of premiR-27a are indicated in bold. (b) A predicted VDR binding site (red) in human *miR-27b* gene promoter. The initial codon (ATG) is indicated in bold. (c-e) ChIP analysis of three VDR binding sites (site 1 (c), site 2 (d), site 3 (e)) located in *miR-27a* in VDR or control plasmids-transfected HOKs with IgG or VDR

antibodies treatment as indicated. (f) ChIP analysis of VDR binding site related with *miR-27b* in VDR or control plasmids-transfected HOKs with antibodies treatments as shown. (g) ChIP assays of VDR plasmids-transfected HOKs with IgG or VDR antibodies treatment. (h) SNAP25 and TXN2 levels in HOKs transfected with control or VDR plasmids tested by qPCR. *P < 0.05, **P < 0.01, ***P < 0.001 vs. corresponding control; # P < 0.05, ##P < 0.01 vs the third (VDR plasmids and IgG antibody) group; n = 3. Ctrl, control.

Supplemental figure 4

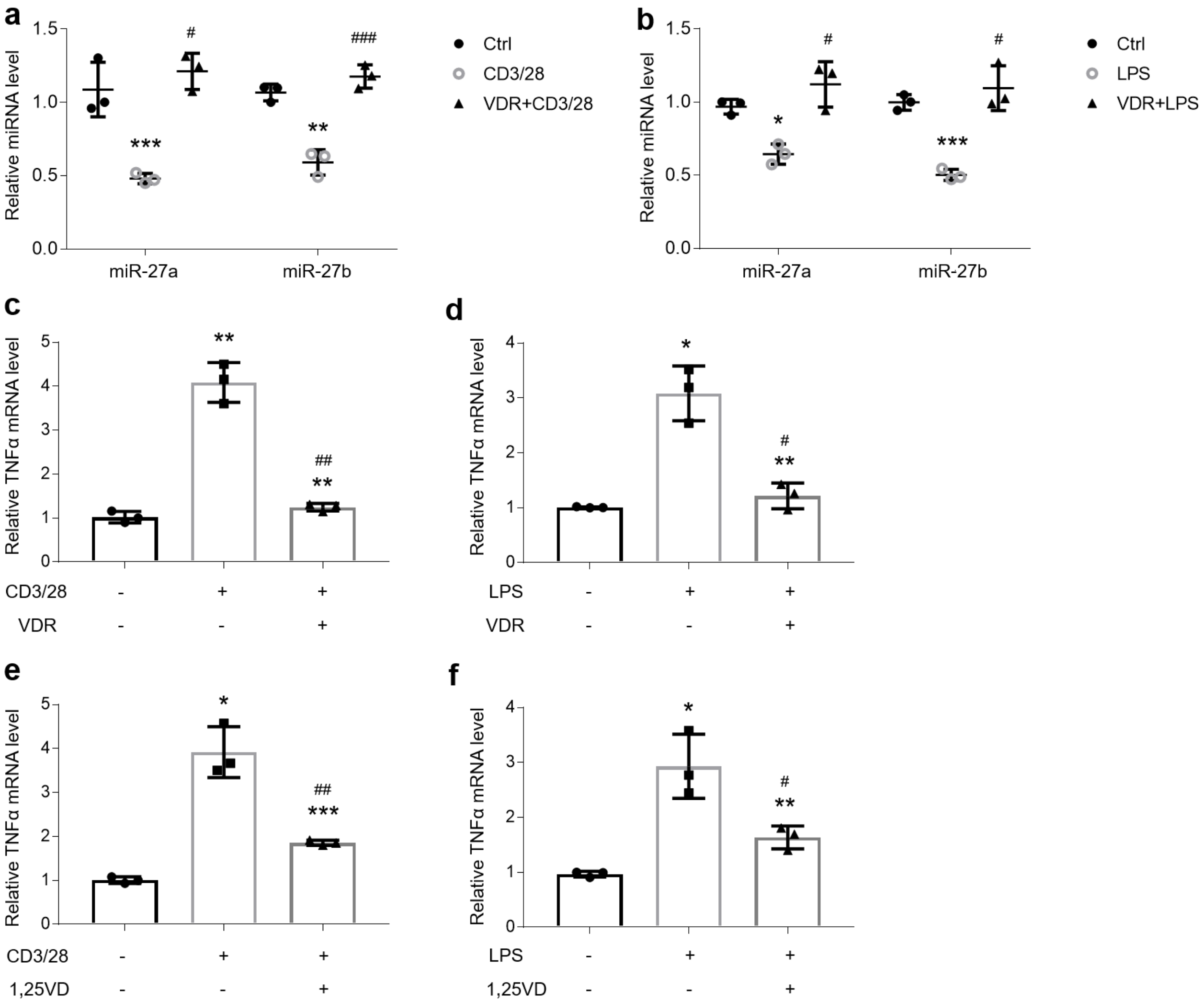

Supplemental figure 4. VDR overexpression rescues miR-27a/b decreases and TNFα increase in OLP cell models. (a and b) Real-time PCR analyses of miR-27a/b status in culture medium of activated CD4$^+$ T cell (a)- or LPS (b)-treated HOKs with or without VDR plasmids transfection. (c and d) Real-time PCR assays of TNFα levels

in activated $CD4^+$ T cell (c)- or LPS (d)-stimulated HOKs with or without VDR plasmids transfection. (e and f) TNFα expression in activated $CD4^+$ T cell (e)- or LPS (f)-treated HOKs with or without 1,25 VD measured by qPCR. *$P < 0.05$, **$P < 0.01$, ***$P < 0.001$ vs. corresponding control; #$P < 0.05$, ##$P < 0.01$, ###$P < 0.001$ vs CD3/28 or LPS group; n = 3. Ctrl, control; 1,25VD, 1,25$(OH)_2D_3$.

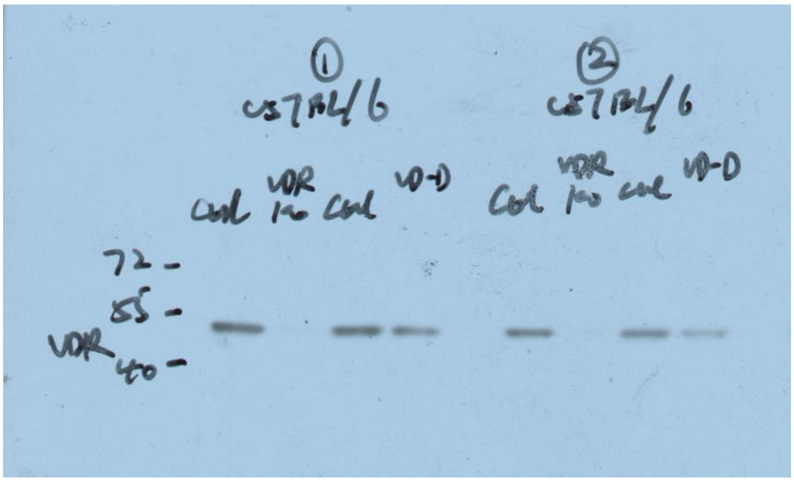

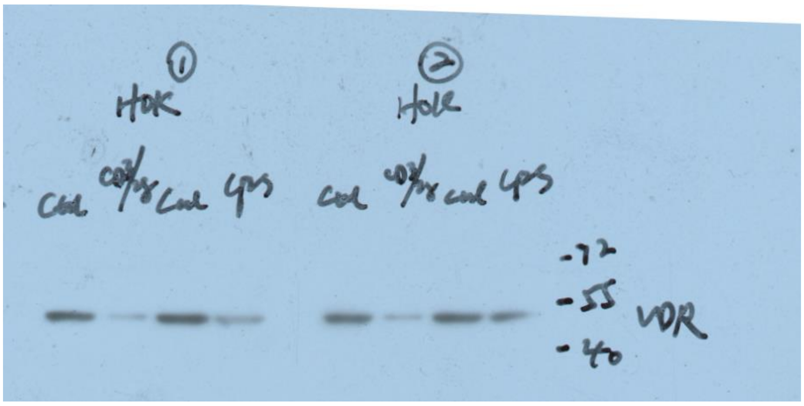

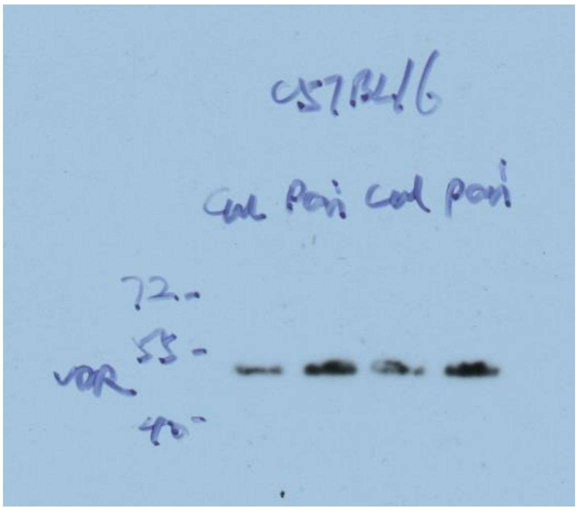

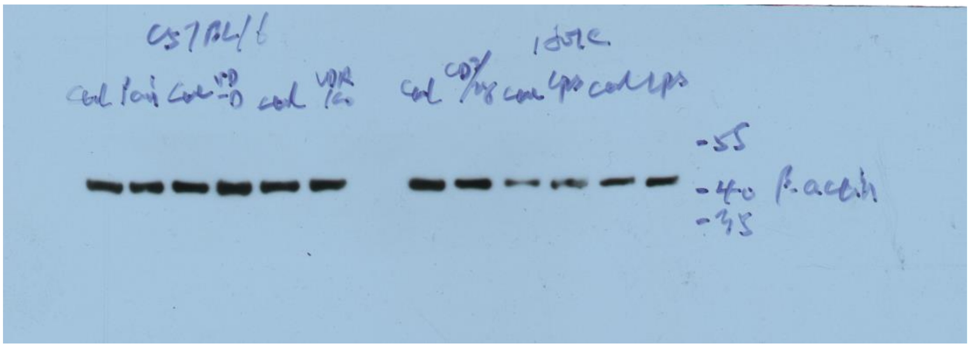
C57BL/6
-55
-40
-35
β-actin
